# Increased Resistance of SARS-CoV-2 Variants B.1.351 and B.1.1.7 to Antibody Neutralization

**DOI:** 10.21203/rs.3.rs-155394/v1

**Published:** 2021-01-29

**Authors:** Pengfei Wang, Lihong Liu, Sho Iketani, Yang Luo, Yicheng Guo, Maple Wang, Jian Yu, Baoshan Zhang, Peter D. Kwong, Barney S. Graham, John R. Mascola, Jennifer Y. Chang, Michael T. Yin, Magdalena Sobieszczyk, Christos A. Kyratsous, Lawrence Shapiro, Zizhang Sheng, Manoj S. Nair, Yaoxing Huang, David D. Ho

**Affiliations:** 1 Aaron Diamond AIDS Research Center, Columbia University Vagelos College of Physicians and Surgeons, New York, NY, USA.; 2 Department of Microbiology and Immunology, Columbia University Irving Medical Center, New York, NY, USA.; 3 Vaccine Research Center, National Institutes of Health, Bethesda, MD, USA.; 4 Department of Biochemistry, Columbia University, New York, NY, USA.; 5 Division of Infectious Diseases, Department of Internal Medicine, Columbia University Vagelos College of Physicians and Surgeons, New York, NY, USA.; 6 Regeneron Pharmaceuticals, Inc., Tarrytown, NY. USA.; 7 Zuckerman Mind Brain Behavior Institute, Columbia University, New York, NY, USA.

## Abstract

The Covid-19 pandemic has ravaged the globe, and its causative agent, SARS-CoV-2, continues to rage. Prospects of ending this pandemic rest on the development of effective interventions. Two monoclonal antibody (mAb) therapeutics have received emergency use authorization^1,2^, and more are in the pipeline^3–6^. Furthermore, multiple vaccine constructs have shown promise^7^, including two with ~95% protective efficacy against Covid-19^8,9^. However, these interventions were directed toward the initial SARS-CoV-2 that emerged in 2019. Considerable viral evolution has occurred since, including variants with a D614G mutation^10^ that have become dominant. Viruses with this mutation alone do not appear to be antigenically distinct, however^11^. Recent emergence of new SARS-CoV-2 variants B.1.1.7 in the UK^12^ and B.1.351 in South Africa^13^ is of concern because of their purported ease of transmission and extensive mutations in the spike protein. We now report that B.1.1.7 is refractory to neutralization by most mAbs to the N-terminal domain (NTD) of spike and relatively resistant to a number of mAbs to the receptor-binding domain (RBD). It is modestly more resistant to convalescent plasma (~3 fold) and vaccinee sera (~2 fold). Findings on B.1.351 are more worrisome in that this variant is not only refractory to neutralization by most NTD mAbs but also by multiple potent mAbs to the receptor-binding motif on RBD, largely due to an E484K mutation. Moreover, B.1.351 is markedly more resistant to neutralization by convalescent plasma (~11–33 fold) and vaccinee sera (~6.5–8.6 fold). B.1.351 and emergent variants^14,15^ with similar spike mutations present new challenges for mAb therapy and threaten the protective efficacy of current vaccines.

SARS-CoV-2 B.1.1.7, also known as 501Y.V1 in the GR clade (Fig. 1a), emerged in September 2020 in South East England and rapidly became the dominant variant in the UK, possibly due to its enhanced transmissibility^12^. This strain has now spread to over 50 countries. B.1.1.7 contains 8 spike mutations in addition to D614G, including two deletions (69–70del & 144del) in NTD, one mutation (N501Y) in RBD, and one mutation (P681H) near the furin cleavage site (Fig. 1b). SARS-CoV-2 B.1.351, also known as 501Y.V2 in the GH clade (Fig. 1a), emerged in late 2020 in Eastern Cape, South Africa (SA)^13^. This variant has since become dominant locally, raising the specter that it too has enhanced transmissibility. B.1.351 contains 9 spike mutations in addition to D614G, including a cluster of mutations (e.g., 242–244del & R246I) in NTD, three mutations (K417N, E484K, & N501Y) in RBD, and one mutation (A701V) near the furin cleavage site (Fig. 1b). There is a growing concern that these new variants could impair the efficacy of current mAb therapies or vaccines, because many of the mutations reside in the antigenic supersite in NTD^16,17^ or in the ACE2-binding site (also known as the receptor-binding motif—RBM) that is a major target of potent virus-neutralizing antibodies. We therefore addressed this concern by creating VSV-based SARS-CoV-2 pseudoviruses that contain each of the individual mutations as well as one with all 8 mutations of the B.1.1.7 variant (UKΔ8) and another with all 9 mutations of the B.1.351 variant (SAΔ9). A total of 18 mutant pseudoviruses were made as previously described^18,19^, and each was found to have a robust titer (Extended Data Fig. 1) adequate to measure its susceptibility to neutralization by 30 mAbs, 20 convalescent plasma, and 22 vaccinee sera.

## Monoclonal antibodies

**Fig. 2 |:**
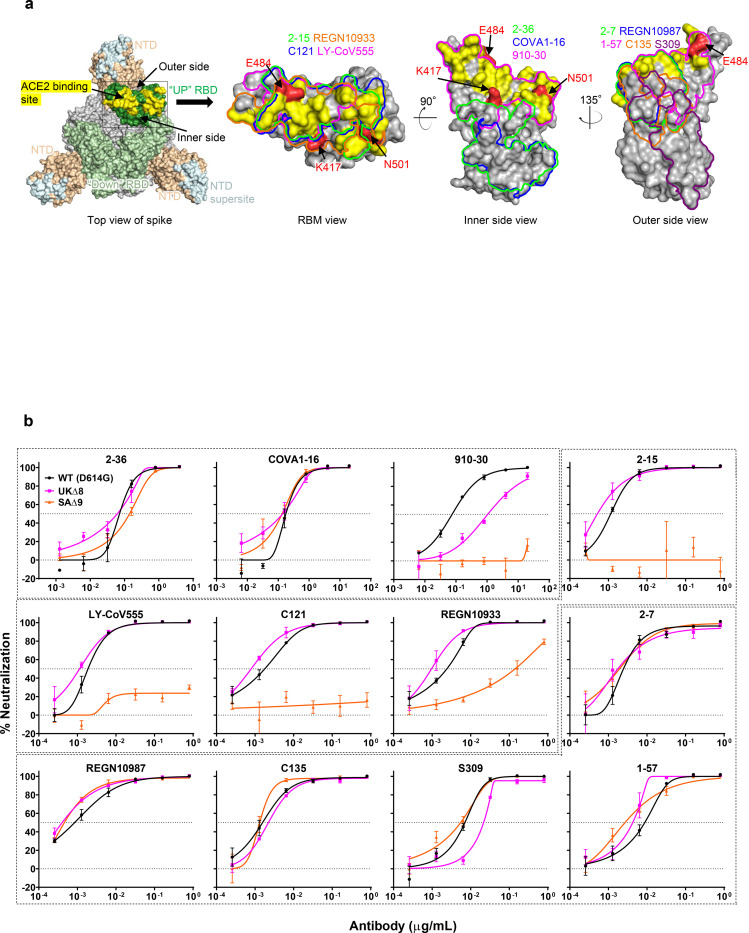

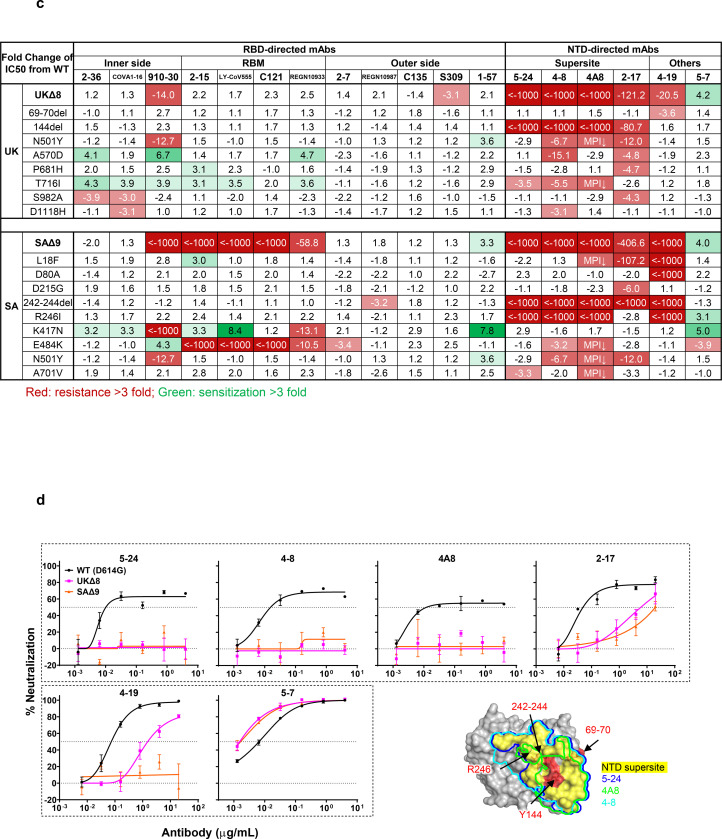

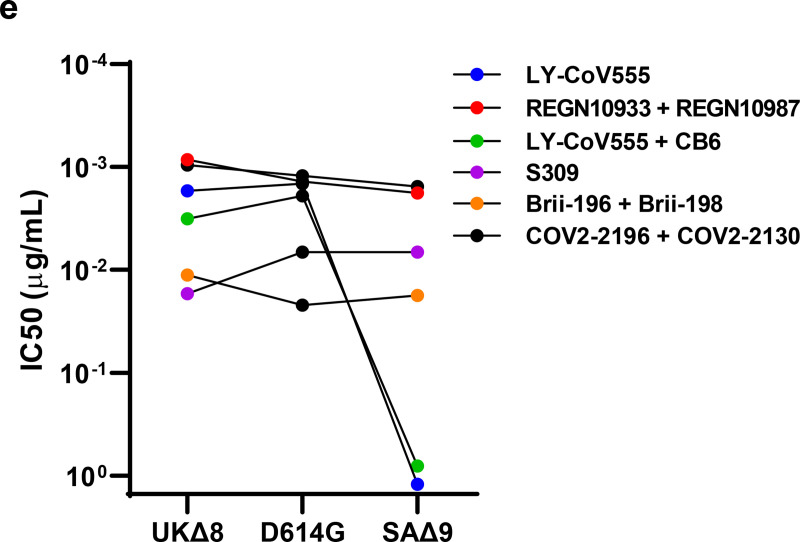
Susceptibility of UKΔ8 and SAΔ9 pseudoviruses to neutralization by mAbs. **a,** Footprints of neutralizing mAbs on the RBD. Left panel, top view of SARS-COV-2 spike with one RBD in the “up” conformation (pdb: 6zgg). RBD and NTD are colored green and peach, respectively. The positions of ‘inner’ and ‘outer’ sides are indicated on the “up” RBD with the ACE2-binding site colored yellow. The three panels to the right show the antibody footprints on RBD. **b,** Neutralization of UKΔ8, SAΔ9, and WT pseudoviruses by select RBD mAbs. **c,** Fold-change in IC50 of neutralizing mAbs against UKΔ8 and SAΔ9, as well as single-mutation pseudoviruses, relative to WT. MPI↓ denotes that maximum percent inhibition is substantially reduced, confounding IC50 calculations. **d,** Neutralization of UKΔ8, SAΔ9, and WT pseudoviruses by NTD-directed mAbs, the footprints of which are delineated by the color tracings in the insert. **e,** Changes in neutralization IC50 of authorized or investigational therapeutic mAbs against UKΔ8 and SAΔ9. Data in **b** and **d** are mean ± SEM of technical triplicates, and represent one of two independent experiments.

We first assayed the neutralizing activity of 12 RBD mAbs against UKΔ8, SAΔ9, and WT (D614G) pseudoviruses in Vero E6 cells as previously described^18,19^. Three mAbs target the “inner side”, four target RBM, and five target the “outer side”. The footprints of these mAbs on RBD are shown in Fig. 2a, and their neutralization profiles are shown in Fig. 2b. For neutralization of UKΔ8, only the activities of 910–30^20^ and S309^4^ are impaired, albeit modestly. For neutralization of SAΔ9, however, the activities of 910–30, 2–15^18^, LY-CoV555 (bamlanivimab)^1,21^, C121^22^, and REGN10933 (casirivimab)^2^ are completely or markedly abolished. The four mAbs that target RBM are among the most potent SARS-CoV-2-neutralizing antibodies in clinical use or development. Note that mAbs directed to lower aspects of the “inner side” (2–36^18^ & COVA1–16^23,24^) or to the “outer side” retain their activities against SAΔ9, including 2–7^18^, REGN10987 (imdevimab)^2^, C135^22^, and S309 that are in clinical use or development. The results on the neutralization of UKΔ8 and SAΔ9 by these 12 mAbs are summarized in Fig. 2c as fold changes in IC50 neutralization titers relative to the WT. To understand the specific spike mutations responsible for the observed changes, we also tested the same panel of mAbs against pseudoviruses containing only a single mutation found in B.1.1.7 or B.1.351. The results are displayed, among others, in Extended Data Fig. 2 and summarized in Fig. 2c. Against UKΔ8, the decreased activity of 910–30 is mediated by N501Y, whereas the slightly impaired activity of S309 is unexplained. Against SAΔ9, the complete loss of activity of 2–15, LY-CoV555, and C121 is mediated by E484K; the complete loss for 910–30 is mediated by K417N; and the marked reduction for REGN10933 is mediated by K417N and E484K. A structural explanation on how E484K disrupts the binding of 2–15, LY-CoV555, and REGN10933 is presented in Extended Data Fig. 3a.

We also assessed the neutralizing activity of six NTD mAbs against UKΔ8, SAΔ9, and WT pseudoviruses. Both UKΔ8 and SAΔ9 are profoundly resistant to neutralization by our antibodies 5–24, 4–8, 2–17, and 4–19^18^, as well as by 4A8^25^ (Fig. 2d). Note that 5–24, 4A8, and 4–8 are known to target the antigenic supersite in NTD^16^ (Insert in Fig. 2d). The activity of 5–7^18^ remains intact, however. To understand the specific mutations responsible for the observed changes, we then tested these mAbs against pseudoviruses containing only a single mutation found in B.1.1.7 or B.1.351 (Extended Data Fig. 2). The results are summarized in Fig. 2c as fold change relative to the WT. It is evident that the resistance of UKΔ8 to most NTD mAbs is largely conferred by 144del, whereas the resistance of SAΔ9 is largely conferred by 242–244del and/or R246I. Amino-acid residues 144, 242–244, and 246 all fall within the NTD supersite^16,17^ (Insert in Fig. 2d; details in Extended Data Fig. 3b). The obvious exception is 5–7, whose neutralizing activity is actually enhanced. Needless to say, a detailed structural understanding of how 5–7 binds NTD will be important.

We next tested the neutralizing activity of 12 additional RBD mAbs, including ones from our own collection (1–20, 4–20, 2–4, 2–43, 2–30, & 2–38)^18^ as well as CB6^5^, COV2–2196 & COV2–2130^6^, Brii-196 & Brii-198^3^, and REGN10985. The results against UKΔ8, SAΔ9, and WT are highlighted in Extended Data Fig. 4a, and the detailed findings against the single-mutation pseudoviruses are shown in Extended Data Fig. 2. The fold changes in neutralization IC50 titers relative to the WT are tabulated in Extended Data Fig. 4b. Herein we only comment on results for mAbs in clinical development. The activity of CB6 is slightly impaired against UKΔ8, likely due to N501Y and/or S982A, but it is rendered inactive against SAΔ9 because of K417N. Brii-196 and COV2–2130 are essentially unaffected by the new variants; the activities of Brii-198 and COV2–2196 are slightly diminished against SAΔ9 but not against UKΔ8.

Lastly, we examined, in a single experiment, the neutralizing activity of mAb therapies in clinical use or under clinical investigation against UKΔ8, SAΔ9, and D614G pseudoviruses. The results for single mAbs LY-CoV555 and S309, as well as for combination regimens REGN10933+REGN10987, LY-CoV555+CB6, Brii-196+Brii-198, and COV2–2196+COV2–2130, are shown in Extended Data Fig. 5 and summarized in Fig. 2e. Note that LY-CoV555, alone or in combination with CB6, is no longer able to neutralize SAΔ9. While REGN10933+REGN10987 and COV2–2196+COV2–2130 are seemingly unaffected, each of these potent combinations has a component that has lost some neutralizing activity (Fig. 2c & Extended Data Fig. 4b). Although S309 and the Brii-196+Brii-198 combination are not significantly impaired, their potencies are noticeably lower (Fig. 2e). These findings suggest that antibody treatment of this virus might need to be modified in localities where B.1.351 and related variants^14,15^ are prevalent, and highlight the importance of combination antibody therapy to address the expanding antigenic diversity of SARS-CoV-2.

## Convalescent plasma

We obtained convalescent plasma from 20 patients more than one month after documented SARS-CoV-2 infection in the Spring of 2020. Ten had severe disease and 10 had non-severe disease, as previously defined^19^. Their ages ranged from 34 to 79, with a mean of 54. Six were female, and 14 were male.

**Fig. 3 |:**
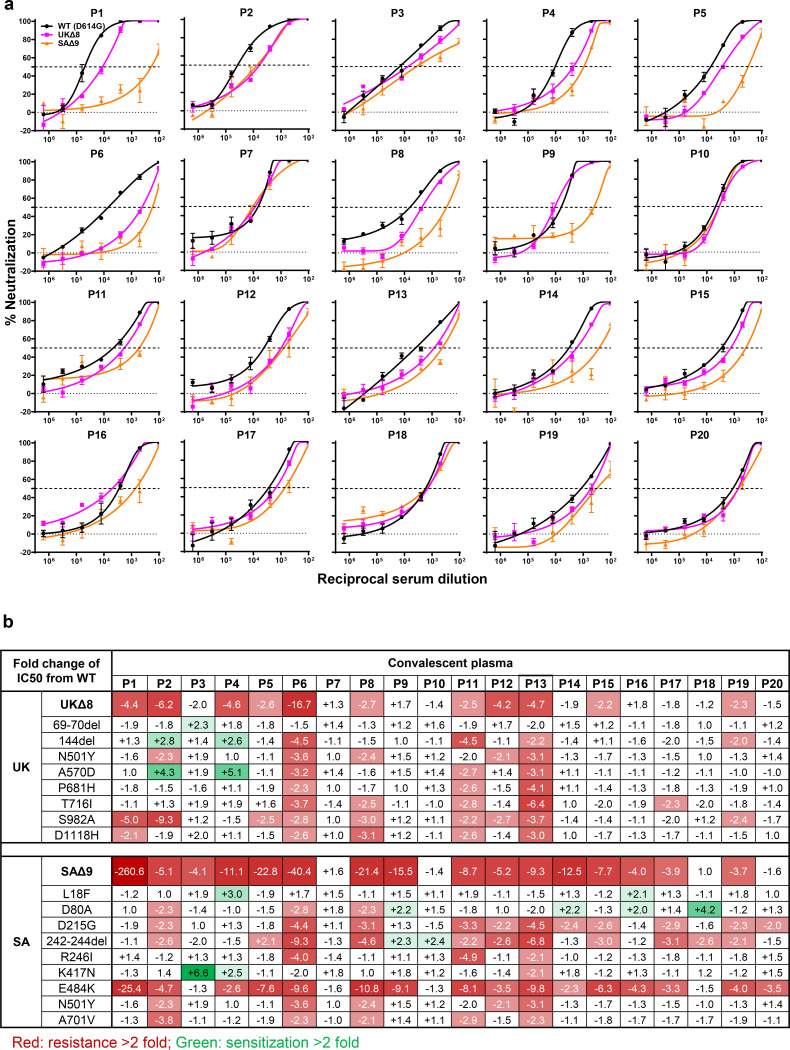

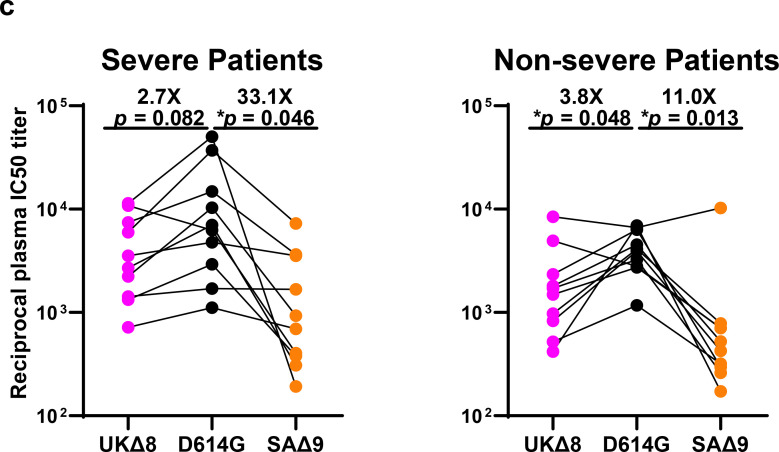
UKΔ8 and SAΔ9 pseudoviruses are more resistant to neutralization by convalescent plasma from patients. **a,** Neutralization results for 20 convalescent plasma samples (P1-P20) against UKΔ8, SAΔ9, and WT. Data represent mean ± SEM of technical triplicates. The panels are arranged by IC50 values against the WT, from low to high. **b,** Fold change in neutralization IC50 of UKΔ8 and SAΔ9, as well as single-mutation pseudoviruses, relative to the WT presented as a heatmap with darker colors implying greater change. **c,** Change in reciprocal plasma neutralization IC50 values of convalescent plasma from severe and non-severe patients against UKΔ8 and SAΔ9, relative to the WT. Mean fold changes in IC50 values relative to the WT are written above the *p* values. Statistical analysis was performed using a two-tailed paired *t* test.

Each plasma sample was then assayed for neutralization against UKΔ8, SAΔ9, and WT pseudoviruses. Fig. 3a shows that most plasma samples lost >2-fold neutralizing activity against the new variants relative to the WT. The loss in potency is more frequent against SAΔ9 (16 of 20) than against UKΔ8 (11 of 20). Only plasma from P7, P10, P18, and P20 retain neutralizing activities identical or similar to those against the WT. These results are summarized as fold change in plasma neutralization IC50 titers in Fig. 3b. Furthermore, the magnitude of the drop in plasma neutralization is better seen in Fig. 3c, with the overall mean loss of activity being modest against UKΔ8 (2.7 to 3.8 fold), but more substantial against SAΔ9 (11.0 to 33.1 fold).

Every plasma sample was also tested against each single-mutation pseudovirus, and those findings are shown in Extended Data Fig. 6 and summarized in Fig. 3b. Unlike the data for mAbs (Fig. 2c), no single mutation could predictably account for the loss of plasma neutralizing activity against UKΔ8, indicating that the mutations in this variant do not perturb an immunodominant epitope on the spike that is shared by many infected persons. S982A seems to have a discernible negative impact on the plasma neutralizing activity of 9 samples (Fig. 3b), perhaps due to its interaction with the bottom of RBD (Extended Data Fig. 3c). On the other hand, the loss of plasma neutralizing activity against SAΔ9 could be largely attributed to E484K (Fig. 3b), suggesting that this RBM mutation to be situated in an immunodominant epitope for most infected persons. It is also interesting to note that cases such as P7 and P10 have neutralizing antibodies that are essentially unperturbed by the multitude of spike mutations found in these two new variants (Fig. 3b). A detailed analysis of their antibody repertoire against the viral spike could be informative.

## Vaccinee Sera

Sera were obtained from 12 participants of a Phase 1 clinical trial of Moderna SARS-Co-2 mRNA-1273 Vaccine^8^ conducted at the NIH. These volunteers received two immunizations with the vaccine (100 μg) on days 0 and 28, and blood was collected on day 43. Additional vaccinee sera were obtained at Columbia University Irving Medical Center from 10 health care workers who received the Pfizer BNT162b2 Covid-19 Vaccine^9^ at the clinical dose on days 0 and 21. Blood was collected on day 28 or later.

**Fig. 4 |:**
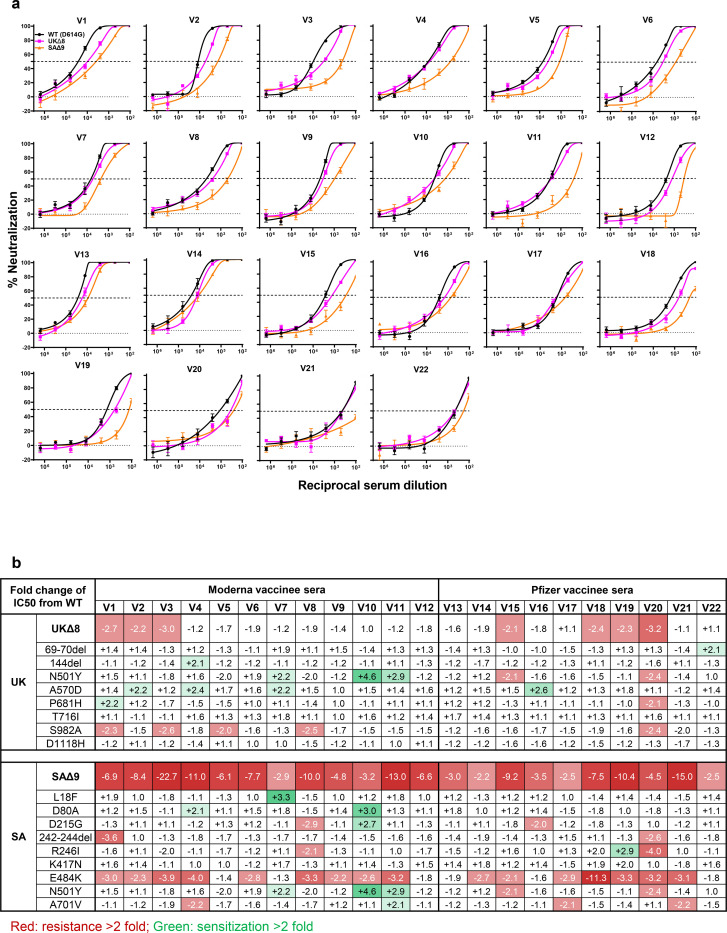

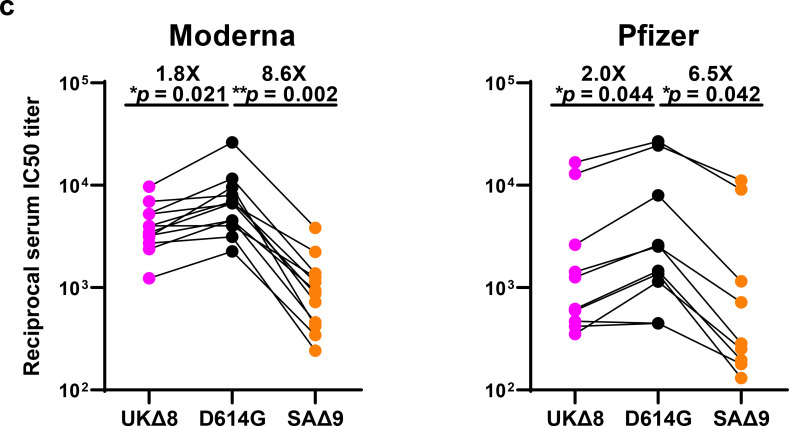
UKΔ8 and SAΔ9 pseudoviruses are more resistant to neutralization by vaccinee sera. **a,** Neutralization profiles for 22 serum samples obtained from persons who received SARS-CoV-2 vaccine made by Moderna (V1-V12) or Pfizer (V13-V22) against UKΔ8, SAΔ9, and WT pseudoviruses. The panels are arranged by IC50 values against the WT, from low to high for each set of vaccinees. Data are mean ± SEM of technical triplicates, and represent one of two independent experiments. **b,** Fold change in serum neutralization IC50 of UKΔ8 and SAΔ9, as well as single-mutation pseudoviruses, relative to the WT presented as a heatmap with darker colors implying greater change. **c,** Change in reciprocal serum IC50 values for Moderna and Pfizer vaccinees against UKΔ8 and SAΔ9, relative to the WT. Mean fold change in IC50 relative to the WT is written above the *p* values. Statistical analysis was performed using a two-tailed paired *t* test.

Each vaccinee serum sample was assayed for neutralization against UKΔ8, SAΔ9, and WT pseudoviruses. Fig. 4a shows only a minority of sera to have lost >2-fold neutralizing activity against UKΔ8, whereas every sample lost activity against SAΔ9, ranging from slight to substantial. These results are quantified and tabulated as fold change in neutralization IC50 titers in Fig. 4b, and the extent of the decline in neutralization activity is more evident in Fig. 4c. Overall, the mean loss of neutralizing activity against UKΔ8 appears to be small (1.8 fold, Moderna; 2.0 fold, Pfizer), but quite significant against SAΔ9 (8.6 fold, Moderna; 6.5 fold, Pfizer).

Every vaccinee serum was also tested against each single-mutation pseudovirus, and the results are presented in Extended Data Fig. 7 and summarized in Fig. 4b. As was the case for convalescent plasma (Fig. 3b), no single mutation could predictably account for the small loss of serum neutralizing activity against UKΔ8. Again, S982A seems to have a minor negative impact on the plasma neutralizing activity of every serum sample (Fig. 4b), possibly due to distal effects on the RBD (Extended Data Fig. 3c). The loss of neutralizing activity against SAΔ9 in vaccinee sera could be principally attributed to E484K (Fig. 4b), indicating that this RBM mutation to be situated in an immunodominant epitope recognized by all vaccinees studied. Our findings do not reveal any significant differences between the two different vaccines.

## Discussion

Both SARS-CoV-2 variants B.1.1.7 and B.1.351 are raising concerns not only because of their increase transmissibility but also because of their extensive mutations in spike that could lead to antigenic changes detrimental to mAb therapies and vaccine protection. It is of equal concern that another variant, B.1.1.28 or 501Y.V3, is increasing rapidly in Brazil and spreading far beyond^14,15^. B.1.1.28 contains three mutations (K417T, E484K, and N501Y) at the same RBD residues as B.1.351. Much of our findings on SAΔ9 would likely be similar for this emergent variant. N501Y is shared among viruses in these three lineages; while this mutation may confer enhanced binding to ACE2^26^, its antigenic impact is limited to a few mAbs (Fig. 2c & Extended Data Fig. 4b), with no pronounced effects on the neutralizing activity of convalescent plasma or vaccinee sera (Figs. 3b & 4b).

Our findings have relevance to the use of mAb to treat or prevent SARS-CoV-2. Both UKΔ8 and SAΔ9 are resistant to neutralization by mAbs directed to the NTD supersite (Figs. 2c, 2d, & Extended Data Fig. 3b). More importantly, SAΔ9 is resistant to a major group of potent mAbs that target the RBM, including two authorized for emergency use (Fig. 2c). LY-CoV555 is inactive against SAΔ9, and the ~60-fold loss in potency of REGN10933 renders the combination of REGN10933+REGN10987 to be effectively monotherapy. Several other mAbs in development are similarly impaired (Figs. 2c, 2e, & Extended Data Fig. 4b) against this variant. Decisions on the use of these mAbs will depend heavily on the local prevalence of B.1.351 or B.1.1.28, thus highlighting the importance of viral genomic surveillance worldwide and proactive development of next-generation antibody therapeutics.

Convalescent plasma from patients infected with SARS-CoV-2 from early in the pandemic show slightly decreased neutralizing activity against UKΔ8, but the diminution against SAΔ9 is remarkable (Figs. 3b &3c). This relative resistance is largely due to E484K, a mutation shared by B.1.351 and B.1.1.28^13–15^. Again, in areas where such viruses are common, one would have heightened concerns about re-infection, which has already been well documented even in the absence of antigenic changes^27,28^.

As for the ramifications of our findings for the protective efficacy of current SARS-CoV-2 vaccines, the ~2-fold loss of neutralizing activity of vaccinee sera against UKΔ8 is unlikely to have an adverse impact due to the large “cushion” of residual neutralizing antibody titer (Fig. 4c). On the other hand, the loss of ~6.5–8.6 fold in activity against SAΔ9 is more worrisome, although the clinical implication for vaccine efficacy remains to be determined. The results from ongoing trials in South Africa using these or similar vaccine constructs should be informative.

The recent emergence of B.1.1.7, B.1.351, and B.1.1.28 is a clear demonstration of SARS-CoV-2 antigenic drift. This conclusion is supported by data presented herein, illustrating how so many of these spike changes conferred resistance from antibody neutralization. Mutationally, this virus is traveling in a direction that could ultimately lead to escape from our current therapeutic and prophylactic interventions directed to the viral spike. If the rampant spread of the virus continues and more critical mutations accumulate, then we may be condemned to chasing after the evolving SARS-CoV-2 continually, as we have long done for influenza virus. Such considerations require that we stop virus transmission as quickly as is feasible, by redoubling our mitigation measures and by expediting vaccine rollout.

## Supplementary Material



## Figures and Tables

**Fig. 1 |:**
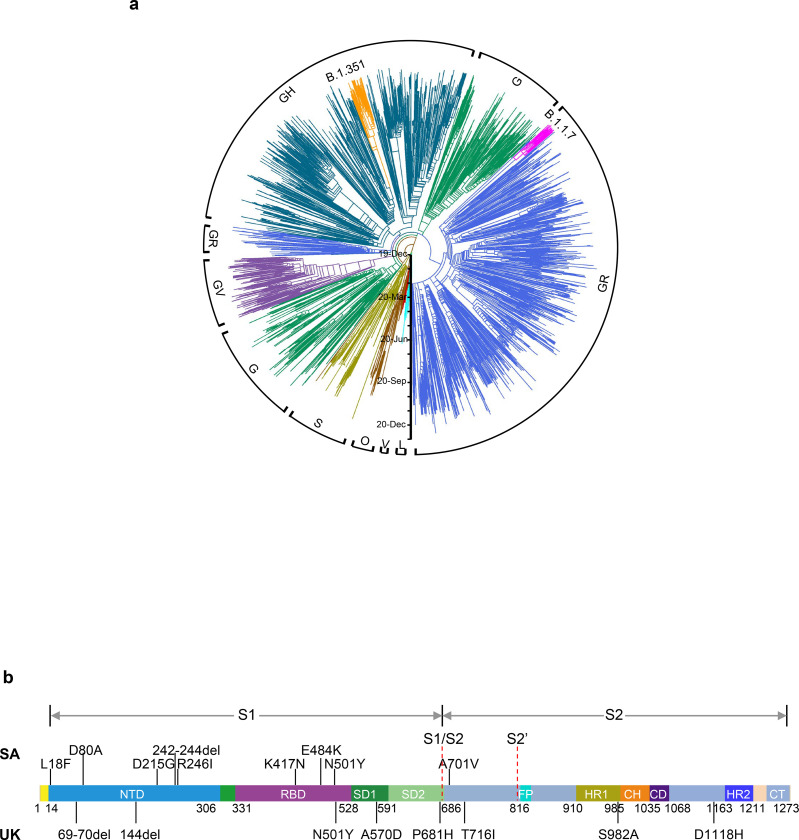
Emerging SARS-CoV-2 variants identified in the UK and SA. **a,** Phylogenetic tree of SARS-CoV-2 variants, with B.1.351 and B.1.1.7 highlighted. **b,** Mutations in the viral spike identified in B.1.351 (SA) and B.1.1.7 (UK) in addition to D614G.
